# Exploring user experiences of clinicians engaged with the digital healthcare interventions across the referral and university teaching hospitals in Nigeria: a qualitative study

**DOI:** 10.3389/fdgth.2025.1488880

**Published:** 2025-05-29

**Authors:** Uchechukwu Solomon Onyeabor, Okechukwu Onwuasoigwe, Wilfred Okwudili Okenwa, Thorsten Schaaf, Niels Pinkwart, Felix Balzer

**Affiliations:** ^1^ Department of Computer Science, Federal University Oye-Ekiti, Oye-Ekiti, Nigeria; ^2^ Department of Surgery, University of Nigeria Teaching Hospital, Ituku-Ozalla, Nigeria; ^3^ Department of Surgery, ESUT Teaching Hospital Parklane, Enugu, Nigeria; ^4^ Institute of Medical Informatics, Charité – Universitätsmedizin Berlin, Berlin, Germany; ^5^ Institute of Computer Science, Humboldt-Universität zu Berlin, Berlin, Germany

**Keywords:** challenges and limitations, electronic health record adoption, healthcare digital transformation, university teaching hospitals, referral hospitals, clinicians, developing economies, resource-constrained economies

## Abstract

**Introduction:**

Given that Nigeria and several developing countries are still at the early stage of digital healthcare interventions adoption (like the use of electronic health records systems) there is scarcity of research/empirical reports investigating the overall user experiences of clinicians, the doctors and the nurses who are or who had been practically engaged with the use of these new digital healthcare support implementations that had engendered new culture across their care delivery facilities. The referral and university teaching hospitals in Nigeria numbering over 166 and scattered across over 37 states of the federation and the Federal Capital Territory (FCT) make up a strategic component of Nigeria's healthcare ecosystem. This research was therefore designed and restricted to clinicians who had used these systems so as to explore their experiences with these systems and possibly unveil any challenges/limitations that can bedevil successful and sustainable acquisition of digital healthcare intervention programmes and projects across referral and university teaching hospitals in the Southeastern Region of Nigeria; and could also hamper any future implementations.

**Method:**

This study was designed in a manner that allows the clinicians the liberty to conveniently express in writing (via comments) issues, challenges and concerns that they had perceived, encountered or experienced bedevilling electronic health record adoption and use across their care facilities. So a structured interview method was chosen by the research team (after due considerations) as fitting the research context. This (structured interview) was therefore designed and targeted at about 400 clinical participants, including the doctors and the nurses from three select referral and university teaching hospitals in the Southeastern Region of Nigeria (a federal, state, and national specialist referral hospital).

**Result:**

Out of the 400 clinicians who were targeted in the survey, 326 of them practically responded to the interview questions. The outcome showed the clinicians willingly exposing several issues and challenges that had stifled electronic health record adoption across the hospitals. Issues identified were categorized into themes including challenges bordering on lack of political will on the part of hospital administration; lack of computer/digital/EHR literacy; poor and often lack of comprehensive training on the workings of EHR; poor maintenance culture; poor EHR system design, poor implementation and use-based struggles and challenges; infrastructure issues, system breakdown and network challenges etc were reported.

**Conclusion:**

The outcome of this investigation has profoundly exposed practical issues that had hitherto stifled and often suffocated electronic health record implementation projects across referral and university teaching hospitals in Nigeria. And given the strategic importance of these hospitals in Nigeria healthcare ecosystem, conscious and concerted efforts must be made to address them.

## 1 Introduction

In recent decades, the global healthcare landscape has witnessed a paradigm shift towards the digitalization of healthcare services (1), with Electronic Health Record (EHR) systems emerging as a cornerstone of this transformation (2). Healthcare providers rely on electronic health records systems (EHR-Systems) as a crucial tool for keeping track of patient data and promoting collaboration between medical professionals (2). EHRs, defined as comprehensive digital records of patients' medical history, diagnoses, treatments, medications, and other relevant information, promise to revolutionize healthcare delivery by enhancing efficiency, accuracy, and patient care outcomes (3). However, the successful adoption and integration of EHR systems remain elusive in many settings (4–6) particularly in developing economies, including and across the referral and the university teaching hospitals.

Referral and university teaching hospitals play a pivotal role in the healthcare ecosystem of developing economies, serving as hubs for specialized medical care, medical education, and research (7). These institutions cater to a diverse patient population, often handling complex cases and providing advanced medical interventions (7). As such, the effective utilization of EHR systems within these hospitals is crucial for improving healthcare quality, promoting evidence-based practice, and advancing medical research (8, 9).

Despite the potential benefits of EHR systems, its adoption in public referral and university teaching hospitals in developing economies is often fraught with challenges and limitations (5, 8, 10); such challenges can range from technical issues, such as the compatibility of different systems and software, to socio-cultural barriers including resistance to change and lack of trust in technology (5, 8, 10).

Now in general, healthcare institutions in developing and resource-constrained economies face unique and daunting challenges that hinder the implementation of digital healthcare technologies, such as electronic health records (EHRs). These challenges include significant technical issues, such as inadequate ICT infrastructure and a lack of technical skills (11, 12). Additionally, the absence of robust data protection and cybersecurity laws exacerbates the risks associated with digital health technologies. Interoperability issues also pose a major hurdle, as achieving seamless data exchange between different healthcare systems is difficult due to the lack of standardized data formats and integration capabilities. Furthermore, navigating the complex regulatory landscape, which often includes conflicting and overlapping regulations, adds another layer of difficulty to the implementation of digital health technologies (11).

Another significant challenge facing many healthcare institutions in developing countries is the lack of funding to support her healthcare digital intervention programmes and projects (13). Many of these institutions operate on tight budgets that are often inadequate to meet the basic healthcare needs of the population. As a result, investing in digital intervention technologies may not be a priority for them. Furthermore, the high cost of acquiring and maintaining these technologies is often beyond the reach of many healthcare institutions in low-income countries (13).

Another challenge is the inadequate infrastructure (11). Many public healthcare facilities in developing countries lack basic infrastructure such as reliable electricity and internet connectivity. This could pose a significant obstacle to the implementation of digital interventions, which require a stable and reliable technology infrastructure to function effectively (11). Without these basic requirements, healthcare institutions may find it difficult to leverage the full benefits of digital interventions.

Even in the privately owned and faith-based university teaching hospitals the story appears similar to that of the public. For instance, there was a recent research conducted and aimed to assess the utilization of electronic health records (EHRs) among nurses and identify associated factors in a faith-based Babcock university teaching hospital in Ilishan, Nigeria (14). Using a sequential explanatory mixed-methods approach, the study included a quantitative survey of 240 nurses and qualitative in-depth interviews with 10 purposively selected participants. Quantitative data were analyzed using Chi-square and logistic regression, while qualitative data were subjected to content analysis. The findings revealed that despite the availability of EHR infrastructure—such as software (62.8%), internet connectivity (84.2%), and desktops (76.3%)—EHR utilization was low (27.3%). Factors significantly associated with EHR use included gender, educational qualification, availability of EHR resources, and sponsored training (14). Key barriers identified were the lack of standardized nursing language in EHR software, absence of institutional enforcement policies, and insufficient training opportunities. The study recommended the implementation of comprehensive EHR packages, continuous sponsored training, and the development of clear policies to enhance EHR adoption and effective utilization among nurses (14).

Finally, on this segment, further challenges reported by some literatures included a shortage of skilled personnel to implement and manage digital interventions in many developing countries (13). Additionally, the lack of training and capacity building opportunities for healthcare workers in these countries further compounds the problem.

All these and many more have remained the bane of electronic health record implementation across healthcare establishments in the developing economies in the particular.

Despite the increasing global trend towards the implementation of Electronic Health Record (EHR) systems aimed at enhancing clinical outcomes, there exists a considerable lack of empirical research from the developing context and Nigeria in particular that examines the experiences and challenges faced by clinicians involved in the adoption and implementation and use of these digital healthcare interventions. This is particularly evident within the complex tertiary healthcare environment of the referral and university teaching hospitals in Nigeria. There is currently scarcity of scholarly publications reporting on the overall user experiences and operational difficulties encountered by clinicians, specifically doctors/physicians and the nurses who are the primary end users of EHR systems.

The afore identified research gaps highlight the necessity for empirical investigation to comprehend the viewpoints, obstacles, and constraints faced by healthcare professionals during the adoption and implementation of Electronic Health Record (EHR) systems in developing nations, especially in tertiary healthcare environments. To address this deficiency, the present study aims to enrich the existing literature by conducting a qualitative analysis that examines the perspectives of clinicians at three designated referral and university teaching hospitals located in Southeastern region of Nigeria. These institutions serve as pivotal components of Nigeria's healthcare framework, where multiple efforts to implement EHR systems have been undertaken. By concentrating on the experiences of clinicians within these facilities, this research aspires to reveal critical insights into the difficulties and challenges linked to the adoption and use of EHR systems in the Nigerian healthcare landscape. The anticipated findings of this study are expected to yield evidence-based insights that can enhance current EHR systems and facilitate future implementations in analogous healthcare environments. By acquiring a more profound understanding of the barriers and limitations encountered by clinicians, stakeholders such as healthcare administrators, policymakers, and technology developers can formulate targeted strategies to improve the adoption, usability, and overall effectiveness of EHR systems, thereby enhancing healthcare delivery and patient outcomes in Nigeria and other developing regions. Ultimately, this research seeks to bridge the knowledge gap and foster the successful integration of EHR systems within the healthcare systems of developing economies.

## 2 Methods

### 2.1 The research setting

The qualitative study took place at three hospitals in the Southeastern region Nigeria: FUTH, SUTH, and NSTH. FUTH, a federal university teaching hospital, offered a wide range of specialized healthcare services across disciplines like orthopaedic medicine, radiology, and surgery. It also conducted training programs such as residency training and nursing school programs. The SUTH, a state university teaching hospital, served as a referral medical facility and training ground for the state university college of medicine. It provides specialized care in general medicine, obstetrics, paediatrics, and surgical specialties. Additionally, it offered services like pharmacy, laboratory examinations, and radiology onsite.

#### 2.1.1 Hospital capacities

FUTH had a capacity of over 500 beds and played a significant role in medical education and training. SUTH, with a capacity to accommodate approximately 350–400 patients, was established and designated by the state government. NSTH, a national specialist referral center, was renowned for its orthopaedic medical services and had a long-standing history in providing expert care and training in various medical fields. It strategically catered to the healthcare demands of different regions.

Meanwhile, to obtain the above information, the research team engaged with hospital personnel to gather detailed information about the healthcare infrastructure and services provided by each institution.

### 2.2 The research Aim

This research aims to investigate the challenges and limitations bedevilling EHR-Systems adoption across the referral and university teaching hospitals in the developing economies.

### 2.3 The research design

The research team for this study comprised of about seven personnel, including the principal investigator, three consultant orthopaedic doctors, who held key academic positions within the medical schools of the hospitals under investigation. The following activities were performed:

#### 2.3.1 Data collection and ethical considerations

Data collection involved a structured interview targeting approximately 400 clinical respondents, including doctors and nurses, from three selected tertiary hospitals in the Southeastern region of Nigeria. The research team distributed structured interviews physically, utilizing both manual distribution methods and coordination through heads of clinical units to ensure comprehensive representation of perspectives. Prior to the questionnaire distribution, the team conducted thorough observations of hospital circumstances and engaged stakeholders to gather insights into their experiences, ensuring that the interview questions were relevant and reflective of the challenges faced in EHR adoption. The structured interview questions allowed clinicians to express their issues, challenges, and concerns regarding EHR systems, ultimately leading to a robust dataset for analysis. The team developed the four key open-ended interview questions, based on their observations.

Following the data collection phase, feedback and comments provided by clinicians were meticulously collected, analysed, and categorized into themes that addressed critical components of healthcare digital transformation.

The cooperation and collaboration with the heads of different clinical units played a pivotal role in facilitating access to a diverse range of perspectives and experiences from the clinicians.

Ethical approval for the research was obtained from the Ethical Committees of the hospitals prior to the commencement of the study, ensuring adherence to ethical standards and guidelines. This methodological approach, guided by the COREQ framework, aimed to provide a robust foundation for understanding the challenges and limitations surrounding Electronic Health Record (EHR) system adoption in the context of Nigerian healthcare settings. One may refer to the COREQ checklist uploaded as a Supplementary File to this manuscript for details on the procedures followed in this research.

#### 2.3.2 Sampling technique

The sample selection was deliberately designed to ensure a diverse representation of healthcare professionals directly involved in patient care and medical services within the institutions. The authors targeted approximately 400 clinicians, including doctors and nurses, who were end-users of healthcare digital transformation initiatives, to capture first-hand experiences regarding challenges faced in EHR adoption. The participants were randomly selected and the study tool physically distributed to the participants based on specific criteria, including that they are actively involved in patient care and also familiar with any existing digital healthcare systems in their facility. This is to ensure that their responses stemmed from a comprehensive understanding of the issues surrounding EHR implementation in the Nigerian healthcare context.

Meanwhile, the choice of a sample size of 400 clinicians was guided by the need to ensure adequate representation of doctors and nurses across the selected federal, state, and national specialist teaching hospitals. The total population (*N*) of clinicians in these hospitals includes 556 doctors and 576 nurses at the Federal University Teaching Hospital (FUTH), 379 doctors and 570 nurses at the State University Teaching Hospital (SUTH), and 106 doctors and 356 nurses at the National Specialist Teaching Hospital (NSTH), summing up to a total population of 2,543 clinicians. The sample size was determined to capture a diverse range of experiences and challenges related to Electronic Health Record (EHR) adoption while ensuring thematic saturation in qualitative responses. Now, with a confidence level of 95% and a margin of error of 5%, the required sample size would be approximately 334 participants using Cochran's formula for finite populations, which is expressed as follows:

$$\begin{array}{rl} & n_{0}=\frac{Z^{2}p(1-p)}{e^{2}}\\ & n=\frac{n_{0}}{1+(n_{0}-1/N)} \end{array}$$

where:

• *n*_0_ is the initial sample size for an infinite population,
• *Z* is the *Z*-score corresponding to a 95% confidence level (1.96),
• *p* is the estimated proportion of the population with the characteristic of interest (assumed to be 0.5 for maximum variability),
• *e* is the margin of error (0.05),
• *N* is the total population (2,543).

However, to enhance the robustness of the findings and account for potential non-responses, a sample of 400 was targeted. This approach aligns with established sampling methodologies in healthcare research, ensuring that the study findings are both credible and generalizable within the study context.

#### 2.3.3 Structure interview instrument validation

The validation of the structured interview instrument involved a collaborative effort among the research team and clinicians, including senior consultant orthopaedic doctors with academic positions in the hospitals' medical schools. This process included thorough discussions to ensure that the interview questions were relevant and reflective of the prevailing circumstances in the healthcare settings being studied. The outcome of this validation process led to the refinement of the tool, ensuring that it effectively captured the clinicians' experiences and insights regarding the challenges of Electronic Health Record (EHR) adoption, ultimately enhancing the reliability and relevance of the data collected.

#### 2.3.4 Data analysis

The authors conducted a manual thematic analysis to analyze the data collected from the structured interviews, systematically organizing and categorizing clinician feedback into relevant themes. Researchers began by thoroughly reviewing each interview response, highlighting key phrases and concepts that reflected the participants' experiences and challenges with EHR adoption. They then grouped these responses into thematic categories, such as technical issues, training deficiencies, and resistance to change, allowing for a clearer understanding of the prevalent concerns. This manual approach facilitated a nuanced interpretation of the data, enabling the researchers to capture the complexity of clinician experiences and the specific barriers they faced. Ultimately, the analysis culminated in the extraction of meaningful insights that addressed the limitations and obstacles encountered in the implementation of EHR systems within the hospital settings, providing a comprehensive overview of the current state of digital healthcare transformation.

#### 2.3.5 Justification for mapping to the technology acceptance model (TAM)

To systematically analyze the factors influencing EHR adoption, the authors mapped the study's findings to the Technology Acceptance Model (TAM) framework, which categorizes adoption determinants into Perceived Ease of Use (PEOU), Perceived Usefulness (PU), and Additional External Influences. This process involved thematic analysis, where identified challenges from clinicians' responses were grouped into relevant TAM categories based on their impact on usability, perceived benefits, or external adoption barriers. TAM was chosen because it is a well-established model for understanding technology adoption in healthcare, providing a structured way to assess how usability, usefulness, and external factors shape clinicians' willingness to use EHRs. This framework enabled a clear classification of technical issues (e.g., poor system design under PEOU), organizational challenges (e.g., poor management commitment under PU), and behavioural resistance (e.g., reluctance to change under external influences), ensuring a comprehensive interpretation of the findings.

Meanwhile, the structured interview questions used for the study are as contained in Supplementary Data Sheet 1.

## 3 Results

The expected participation rate was approximately 400 clinicians for the structured interview. However, we obtained responses from only 326 participants, yielding an 81.5% response rate. Table 1 provides a breakdown of the respondents' attributes.

**Table 1:** Characteristics of the clinical respondents' population.

Categories	Demographics	*N*	%
**Total**		**326**	**100%**
Gender			
	Male	60	18.4%
	Female	266	81.6%
Age Range of Clinicians (including the doctors and the nurses studied)			
	20 years to 40 years	208	63.8%
	41 years to 61 years	118	36.2%
How long have you been working			
	1 year to 10 years	204	62.6%
	11 years to 20 years	75	23.0%
	21 years to 35 years	47	14.4%
Highest Education			
	High School	1	0.3%
	Diploma	68	20.9%
	Bachelor	223	68.4%
	Masters	25	7.7%
	PhD/Fellowship	9	2.8%
Job Title			
	Doctors	100	30.7%
	Nurses	226	69.3%
Does your work involve direct patient care			
	Yes	325	99.7%
	No	1	0.3%
How often do you use computer systems			
	Daily	140	42.9%
	A few times a week	89	27.3%
	A few times a month	36	11.0%
	A few times a year	26	8.0%
	Never	35	10.7%
How do you rate your computer skill on a scale of 1 to 5: where 1 = Very Poor, 2 = Poor, 3 = Good, 4 = Very Good, 5 = Proficient			
	1: Very Poor	24	7.4%
	2: Poor	42	12.9%
	3: Good	155	47.5%
	4: Very Good	71	21.8%
	5: Proficient	34	10.4%
Do you have any experience using electronic health record			
	No	162	49.7%
	Yes	164	50.3%
Have you ever received any training on Electronic Health Record			
	No	177	54.3%
	Yes	149	45.7%
Do you know about Cloud Technology and how it could support regular online availability of patients' health records for your hospital and also significantly guarantee efficient and secure access to patients EHR?			
	No	179	54.9%
	Yes	147	45.1%
Would you support Cloud Technology if it could guarantee secure and regular online availability of patients HER			
	No	17	5.2%
	Yes	309	94.8%

Further Results from the Structured Interview Responses can be found in Table 2.

**Table 2:** Restructured table based on TAM framework.

TAM category	Key themes	Frequency	Sample quotes
Perceived Ease of Use (PEOU)	Lack of Computer/Digital Literacy	29	*"Lack of sufficient computer skills among the staff population.” (SUTH Doctor 1)*
	Poor and Lack of Comprehensive Training on EHR	27	*"Several clinical staff members are not yet trained on EHR.” (SUTH Doctor 2)*
	Poor System Design, Implementation & Use-based Struggles	56	*"The existing system had a poorly designed data interface, and access to stored data often remained difficult.” (FUTH Doctor 1)*
	Infrastructure: System Breakdown & Network Issues	39	*"Poor and frustrating internet and network speeds were major challenges.” (FUTH Doctor 2)*
Perceived Usefulness (PU)	Lack of Political Will & Poor Management Commitment	42	*"There is this lack of political will to get things done in our facility.” (SUTH Doctor 3)*
	Poor Maintenance Culture	16	*"There had been persistent complaints about faulty computers.” (NSTH Nurse 1)*
	Poor Power Supply	16	*"Power failure and a low-quality computer may cause everything you type to disappear once the light is out.” (NSTH Nurse 2)*
	Excessive Workload vs. Poor Staff Strength	31	*"High doctor-to-patient ratio, which left no time to manage the EHR.” (SUTH Doctor 4)*
Additional External Influences	Resistance to Change	21	*"Some people still don't want to embrace change.” (SUTH Nurse 1)*
	Poor Staff Motivation	4	*"There is a lack of motivation amongst staff.” (SUTH Nurse 2)*
	Poor Sensitization & Awareness	2	*"There has not been any widespread sensitization about EHR from hospital management.” (FUTH Nurse 1)*
	Funding Challenges	3	*"Financial and funding constraints to implement the project.” (FUTH Nurse 2)*
	Lack of IT Support Staff	3	*"Lack of IT personnel to assist in the event of any challenges.” (NSTH Doctor 1)*

Further details can be found in Supplementary Table 1.

TAM, technology acceptance model; FUTH, Federal university teaching hospital; SUTH, state university teaching hospital; NSTH, national specialist teaching hospital.

Meanwhile, the survey engaged 326 clinicians, slightly below the target of 400, yet provided invaluable insights into the barriers hindering the integration of digital technologies in hospital settings. These challenges were categorized into various themes, including insufficient support from hospital management, inadequate knowledge of computer systems, lack of training in EHR utilization, deficient maintenance practices, substandard design and execution of EHR systems, infrastructure and connectivity issues, staff resistance to change, low motivation and awareness among employees, unstable power supply, increased workload due to understaffing, financial constraints, and shortage of technical support personnel in the IT department. Effectively addressing these challenges is crucial for the successful execution of EHR initiatives in referral and academic hospitals. For more detailed information and sample quotations related to each category of findings, please refer to Table 2.

**Figure 1:**
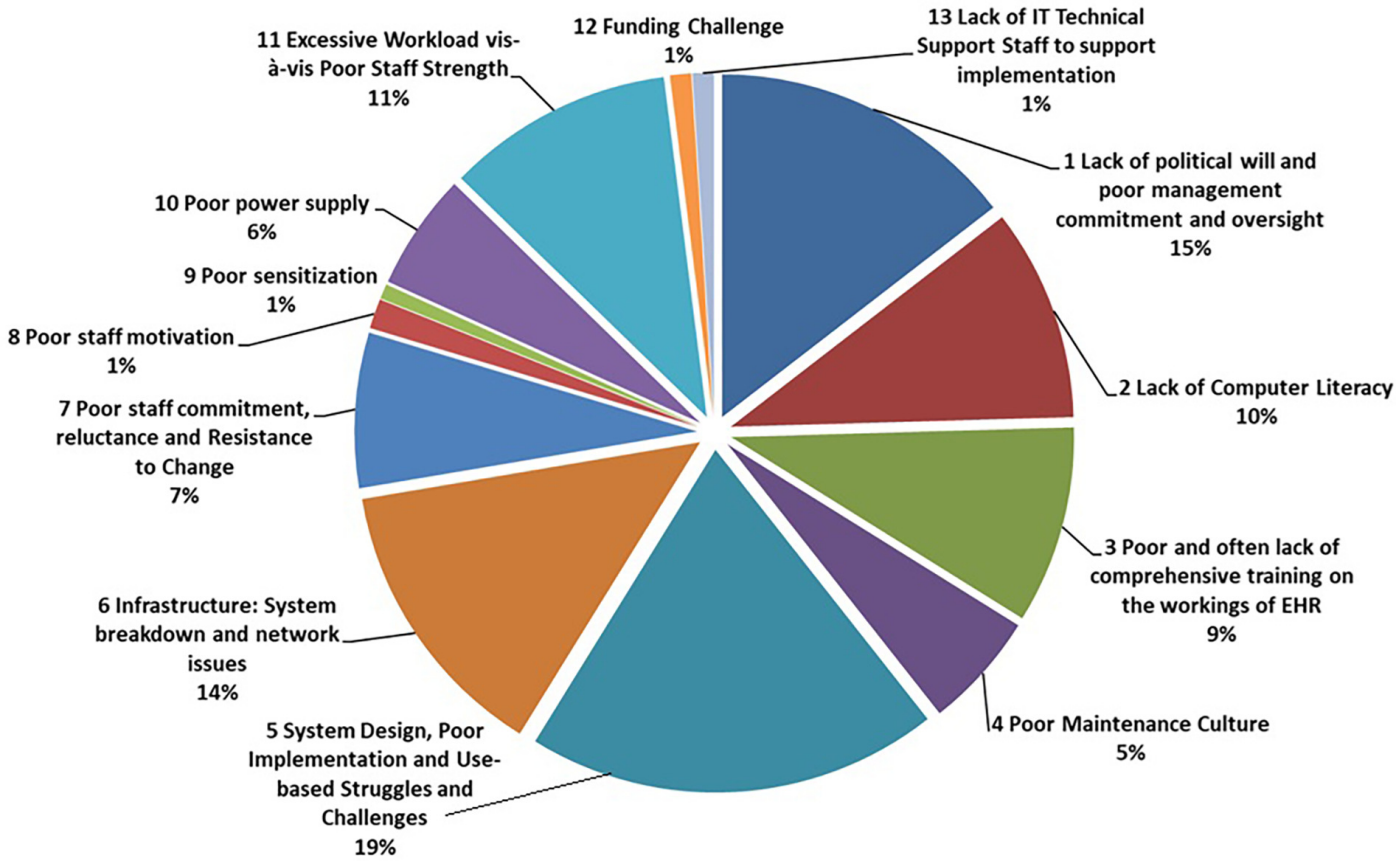
Proposed pie model of clinicians' perceived challenges and limitations bedevilling EHR-system adoption at the referral and the university teaching hospitals in Nigeria.

Meanwhile Figure 1 represents the proposed *Pie Model* of clinicians' perceived challenges and limitations bedevilling EHR-System adoption at the referral and the university teaching hospitals in Nigeria.

### 3.1 Interpretation of result using TAM framework

The use of the Technology Acceptance Model (TAM) was to provide a structured approach to understanding the adoption of Electronic Health Records (EHR) by the clinicians across the target context. It categorized the factors that influence digital healthcare intervention adoption into Perceived Ease of Use (PEOU), Perceived Usefulness (PU), and Additional External Influences. By applying this framework, the study's findings reveal a range of barriers that hinder EHR adoption in Nigerian referral and university teaching hospitals. Table 2 is restructured based on the TAM framework.

#### 3.1.1 Perceived ease of Use (PEOU) factors

A major determinant of EHR adoption is the ease with which clinicians can navigate and use the system. Several participants highlighted lack of computer literacy as a major challenge. As one doctor at SUTH stated, “*Many of our colleagues still struggle with basic computer skills, and typing while attending to patients is a serious challenge.”* Similarly, a nurse at FUTH mentioned, “*Most of the nurses here have never worked with computers before. Using an EHR would require significant training.”* This underscores the need for comprehensive digital literacy programs before EHR systems can be effectively deployed.

The lack of training on EHR systems was another critical issue. A clinician at SUTH remarked, “*We had a one-time training session two years ago, but nothing followed after that. Most of us have forgotten what was taught.”* Another nurse added, “*I was trained on EHR, but the system was never implemented. Now, I don't even remember how to use it.”* Without continuous and practical training, clinicians are unlikely to develop the confidence required to adopt digital records fully.

Furthermore, poor system design and usability issues were repeatedly cited. A doctor at NSTH expressed frustration, stating, “*The system interface is not user-friendly. It takes too long to find patient records, and sometimes, the system crashes mid-session.”* Another participant noted, “*The EHR causes delays in our work. We end up focusing on the computer screen rather than the patient.”* If EHR systems are not designed with clinical workflows in mind, they become a hindrance rather than an enabler of efficiency.

Beyond usability, infrastructure limitations such as network instability and power supply issues pose significant challenges. A clinician at FUTH reported, “*The internet connection is so unreliable that I sometimes spend 20 min just trying to log in.”* Similarly, a nurse at NSTH noted, “*Power failures are a nightmare. We could be in the middle of updating patient records, and suddenly everything goes blank.”* These concerns indicate that technical reliability is crucial for successful adoption. No matter how well an EHR system is designed, it will not be useful if it cannot be accessed consistently due to poor infrastructure and power supply issues.

#### 3.1.2 Perceived usefulness (Pu) factors

Clinicians are more likely to adopt EHRs if they perceive them as beneficial to their workflow and patient care. However, the study revealed that poor hospital management commitment and lack of political will often hinder successful implementation. A doctor at SUTH lamented, “*The hospital announced an EHR project years ago, but no real action has been taken. It's just political talk.”* A nurse at FUTH echoed similar sentiments, stating, “*We were told EHR would be introduced, but after multiple meetings and announcements, nothing has changed.”*

Even when EHRs are introduced, poor maintenance practices often render them ineffective. One doctor at NSTH recounted, “*The computers we use for the EHR system frequently break down, and no one repairs them. It's easier to just go back to paper records.”* Another nurse added, “*We don't even have IT personnel who can assist when the system malfunctions. Most times, we are left stranded.”* Without regular system maintenance and IT support, EHR projects are bound to fail.

Another barrier to perceived usefulness is increased workload due to EHR implementation. A doctor at SUTH expressed concerns, stating, “*Our patient load is already overwhelming. If you add the time-consuming task of entering records into an EHR, it just makes our work harder.”* Similarly, a nurse at NSTH observed, “*The staffing levels here are too low. If you introduce an EHR without increasing the number of workers, we won't be able to cope.”* This highlights the importance of aligning EHR implementation with workforce capacity planning to prevent additional strain on clinicians.

#### 3.1.3 Additional external influences

Beyond usability and perceived benefits, external factors also shape EHR adoption. One of the most cited issues was resistance to change among healthcare staff. A doctor at FUTH noted, “*Many of my colleagues prefer paper-based records. They see no reason to switch to digital.”* Another clinician added, “*EHRs are good, but I'm used to writing notes by hand. It feels faster for me.”* This resistance suggests that change management strategies, such as incentives and gradual transition approaches, are necessary to encourage adoption.

Lack of awareness and poor sensitization further contribute to low adoption rates. A nurse at SUTH explained, “*Many of us don't really know how EHRs work or why we need them. There has been no effort from management to educate us.”* Similarly, another clinician stated, “*We need more seminars and workshops to understand the importance of EHRs. Right now, it feels like just another bureaucratic requirement.”* Hospitals must actively engage clinicians through education campaigns to address skepticism and promote acceptance.

Financial constraints also pose a barrier to implementation. A clinician at NSTH stated, “*We hear about EHRs, but there is simply no budget for it. The hospital cannot afford the necessary infrastructure.”* Another doctor added, *“Funding is a huge problem. Even if we want to adopt EHRs, where will the money come from?”* This emphasizes the need for government funding and private-sector partnerships to support digital healthcare transformation in low-resource settings.

Lastly, the lack of dedicated IT support personnel was identified as a challenge. A nurse at FUTH remarked, “*Whenever there's a system issue, we have no one to turn to. We're expected to figure it out ourselves.”* Similarly, a doctor at NSTH stated, “*Technical support is crucial, but we simply don't have enough trained IT staff to manage the system.”* This suggests that hospitals should invest in dedicated IT teams to support clinicians in the transition to digital records.

In summary, applying the TAM framework reveals that EHR adoption in Nigerian referral and university teaching hospitals is hindered by a combination of usability challenges, doubts about system benefits, and external constraints. Improving ease of use requires targeted training programs, better-designed systems, and reliable infrastructure. Enhancing perceived usefulness involves stronger managerial commitment, improved staffing levels, and well-maintained systems. Additionally, external factors such as resistance to change, financial limitations, and lack of IT support must be addressed to ensure a smooth transition.

To successfully implement EHRs, hospital administrators, policymakers, and technology developers must collaborate on structured interventions that address both technical and human-related barriers. Without this, the full benefits of digital healthcare will remain unrealized, and clinicians will continue to struggle with inefficient and fragmented patient record management.

## 4 Discussion

The research findings have uncovered numerous challenges currently impeding clinical healthcare digital intervention projects in referral and university teaching hospitals in Nigeria. Key themes underscored include the absence of political will and insufficient management commitment and oversight, low levels of computer literacy, digital literacy or EHR literacy among staff, inadequate and often cursory training on healthcare digital interventions, a deficient maintenance culture, substandard system design, implementation issues, and usage-related struggles, infrastructure limitations, staff resistance, and workload concerns. These challenges collectively contribute to the complexities and barriers faced in the successful adoption and integration of Electronic Health Record (EHR) systems within the healthcare landscape of developing economies.

Discussing in details, the lack of political will and poor management commitment and oversight have been highlighted as significant impediments to the effective implementation of electronic health records projects in the hospital settings. These results are consistent with a recent report from Verraki Business Solutions Africa (15), emphasizing the crucial role of political will across the Nigerian government in driving essential reforms within the healthcare sector. This lack of support and leadership buy-in can hinder the progress and sustainability of digital interventions, leading to delays and inefficiencies in healthcare service delivery. Additionally, the low levels of computer/digital/EHR literacy among healthcare staff pose a considerable challenge in utilizing and maximizing the benefits of EHR systems. Without adequate training and support to enhance digital literacy skills, healthcare professionals may struggle to navigate and effectively use digital tools and technologies in their daily practice. This result is consistent with a recent study conducted in Ethiopia, a developing nation, it was found that approximately half of the population sample studied, around 48.2%, had low or quite poor levels of digital literacy among health professionals in referral and teaching hospitals (16).

Moreover, the research findings also shed light on the deficiencies in maintenance culture, system design, and implementation issues that further compound the challenges in healthcare digital transformation projects. Substandard system design and implementation can lead to usability issues, inefficiencies, and user dissatisfaction, impacting the overall success of digital interventions. Existing research indicates that numerous end-users express discontent with the discrepancy between EHR-system designs and clinical workflows, processes, and protocols (17). Furthermore, ineffectively designed systems may lead to cognitive overload and strain, resulting in interference with the patient-physician relationship (18, 19). On the other hand, the lack of proper maintenance practices and infrastructure limitations can also hinder the optimal functioning and sustainability of EHR systems within the hospital settings, creating barriers to seamless healthcare delivery. As per a recent study by Gleiss and Lewandowski (20), hospitals face difficulties in effectively upholding their digital healthcare initiatives due to poor maintenance practices within these establishments.

Furthermore, staff resistance and workload concerns emerged as critical factors influencing the adoption and utilization of EHR systems in the healthcare context. Resistance to change, lack of motivation, and increased workload due to digital interventions can impede staff engagement and participation in the implementation process, affecting the overall effectiveness and acceptance of EHR systems. On resistance to change, Safi et al. (21) emphasized the importance of medical practitioners acknowledging their apprehension towards adopting new technologies or practices. Resistance to change remains a key challenge to effective adoption of digital healthcare supporting technologies. Now addressing these challenges requires a multifaceted approach that includes enhancing leadership support, providing comprehensive training programs on the use of EHR. A recent publication also emphasizes that inadequate education and training may impede the successful integration of electronic health records (22). Again, upgrading infrastructure, managing staff resistance, and optimizing staffing levels to ensure successful digital transformation initiatives in healthcare settings. On infrastructure, Meslamani (11) argues that the insufficiency of infrastructure presents notable technical difficulties that impede the widespread integration of technological resources in healthcare environments within developing countries.

### 4.1 Comparison of key findings from the public hospitals studied vs. Faith-based hospital EHR utilization

Electronic Health Record (EHR) adoption in Nigerian hospitals faces numerous challenges, with variations in utilization rates and barriers between public referral/university teaching hospitals studied and faith-based hospitals. The faith-based hospital studied (14) found that despite the availability of EHR infrastructure—including software (62.8%), internet connectivity (84.2%), and desktop computers (76.3%)—EHR utilization among nurses was still very low at 27.3% (14). Similarly, in public referral and university hospitals studied, clinicians reported limited adoption of EHRs, citing significant challenges that hinder successful implementation. While both healthcare settings experience difficulties in integrating digital health solutions, the nature of these challenges differs due to institutional structures, policies, and administrative frameworks.

One major barrier to EHR adoption in the faith-based hospital was the lack of standardized nursing language in EHR software**,** which made it difficult for nurses to document patient care effectively (14). Additionally, the absence of institutional enforcement policies and insufficient training further contributed to the low usage rates. On the other hand, public hospitals studied faced broader systemic issues, including lack of political will, weak administrative oversight, poor infrastructure maintenance, and staff resistance to digital transition**.** These hospitals also struggled with power outages and unreliable internet connectivity, making it even more difficult to sustain EHR use. The political and bureaucratic complexities in public hospitals studied further exacerbated these challenges, as decision-making processes were often slow and inconsistent.

Another significant factor affecting EHR utilization was training and digital literacy. In the faith-based hospital, nurses who received sponsored training were more likely to use EHRs (14). However, a major issue was the high turnover of trained nurses, leading to a recurring need for retraining. In contrast, the public hospitals studied faced an even greater challenge, as many clinicians had never received formal EHR training, and a significant portion of the workforce lacked basic digital literacy skills. Without adequate technical knowledge, clinicians found it difficult to integrate EHR systems into their daily workflow, leading to frustration and eventual abandonment of digital records in favor of traditional paper-based methods.

The lack of institutional policies and administrative support was another critical issue. In the faith-based hospital, there was no clear institutional policy enforcing the use of EHRs**,** leading to inconsistent adoption among nurses (14). Public hospitals studied, however, suffered from even more severe administrative inefficiencies, where political bottlenecks, lack of commitment from hospital management, and inadequate funding for digital health initiatives stalled EHR implementation. Without strong leadership and structured enforcement, the transition to EHR systems remained incomplete in both settings.

Workload and staffing shortages also played a significant role in hindering EHR adoption. The faith-based hospital reported that staff shortages and high patient volumes made it difficult for nurses to dedicate time to documenting patient records digitally (14). Similarly, public hospitals studied faced a high doctor-to-patient ratio and inadequate staffing, which left clinicians overwhelmed and unable to incorporate EHRs efficiently into their daily routines. The lack of manpower in both settings further discouraged healthcare workers from embracing digital transformation.

In response to these challenges, both studies proposed recommendations to improve EHR adoption. The faith-based hospital study emphasized the need for developing standardized nursing terminologies in EHR software, enforcing institutional policies to mandate EHR usage, and providing continuous training for nurses (14). Meanwhile, the public hospital study suggested stronger political and administrative commitment, increased funding for digital infrastructure, and EHR system designs that align with clinical workflows. Both healthcare settings require a more strategic and structured approach to policy implementation, alongside improved training programs and technological investments, to ensure successful EHR adoption.

Now, while both public and faith-based hospitals struggle with EHR implementation, the public hospitals studied faced more systemic and infrastructural challenges, whereas the faith-based hospital's challenges were policy-related and nursing-specific. Addressing these issues requires targeted interventions**,** such as institutional reforms, better funding for digital infrastructure, continuous capacity building, and stakeholder engagement**.** By overcoming these obstacles, Nigerian hospitals can significantly improve the efficiency and quality of healthcare delivery through effective EHR integration.

Finally the challenge of poor power supply remains a key challenge from the result obtained in this study. The impediment posed by power supply stands out as a prominent and noteworthy factor that serves as a barrier to the effective functioning of institutional establishments within the Nigerian context. In alignment with these observations, Akinseinde (23) drew attention to a series of hurdles encountered during the implementation of EHR-Systems initiatives in Nigeria, encompassing issues such as electricity shortages, limited internet accessibility, and prevailing user perceptions. Consequently, it becomes imperative for Nigeria to confront and effectively address the issue of power supply if it aims to fully optimize and leverage its EHR programs across a spectrum of hospitals situated within the country. Further details graphically illustrated in the *Pie Model*.

### 4.2 Comparison of key findings: Nigeria, Canada, and Germany on EHR adoption

While Nigeria, Canada, and Germany share several challenges in EHR adoption, each country's healthcare landscape presents unique contextual barriers that shape the implementation and effectiveness of digital health records.

#### 4.2.1 Nigeria: infrastructure deficiencies and limited government support

Nigeria faces fundamental challenges in EHR adoption, primarily due to lack of basic infrastructure such as stable electricity, reliable internet connectivity, and sufficient IT support. Many hospitals, particularly in rural and underfunded regions, experience frequent power outages and poor network coverage, making the consistent use of digital health records difficult. In addition, poor system design and limited training opportunities further contribute to the frustration of clinicians, who often struggle with using inefficient or non-user-friendly EHR interfaces. Compounding these issues is a lack of strong government commitment to digital health transformation. Without adequate policy enforcement and sustainable funding, EHR adoption remains slow, and many implementation efforts either fail or remain underutilized.

#### 4.2.2 Canada: fragmented healthcare system and privacy concerns

In Canada, the decentralized nature of healthcare governance presents a significant challenge to EHR implementation. The country's provincial healthcare systems operate independently, leading to inconsistencies in EHR adoption and interoperability across different regions. This lack of national standardization results in fragmented data**-**sharing capabilities, making it difficult for clinicians to access and exchange patient records efficiently. Additionally, privacy and data ownership concerns remain a major issue. Many healthcare stakeholders and patients are uncertain about who controls their health data, which has led to hesitancy in embracing digital records. Despite government support for EHR adoption, the slow progress in policy harmonization and awareness initiatives has limited the widespread use of these systems (24).

#### 4.2.3 Germany: bureaucratic challenges and public skepticism

Germany faces a unique set of challenges primarily driven by a highly fragmented healthcare governance system. The country's self-governing healthcare model has created complex bureaucratic hurdles, slowing down EHR adoption. Medical associations and other stakeholders have historically resisted EHR implementation, contributing to prolonged delays in national rollout efforts. Another major barrier is the public's strong skepticism regarding data security and privacy. Germany has some of the strictest data protection laws in the world, and concerns over patient data misuse or unauthorized access have made many individuals hesitant to adopt electronic records. Furthermore, the complicated application processes for EHR enrollment and lack of user-friendly design have discouraged widespread adoption, leaving the country with one of the lowest EHR usage rates in Europe (25).

By addressing these country-specific challenges through targeted policies, infrastructure investments, and public education efforts, each nation can work toward enhancing its EHR adoption and maximizing the benefits of digital health transformation. Table 3 below contains analysis of policy implications and recommendations between Nigeria, Canada and Germany.

**Table 3:** Policy implications and recommendations.

**Policy recommendation**	**Nigeria**	**Canada**	**Germany**
**Stronger Government Commitment**	National policies must enforce EHR adoption and fund digital health initiatives.	Need for standardized national EHR policies and better data-sharing frameworks.	Policy reforms needed to address governance conflicts and data privacy laws.
**Investment in Digital Literacy & Training**	Comprehensive EHR training programs for clinicians and hospital staff.	Increase patient awareness and healthcare provider support for digital records.	Public education campaigns needed to increase digital health literacy.
**Improving Infrastructure & Interoperability**	Investments in power supply, internet connectivity, and IT support.	Enhance interoperability across provinces to create a unified system.	Improve system integration and cross-border data sharing within the EU.
**Addressing Privacy & Security Concerns**	Develop clear data protection regulations for EHR implementation.	Reevaluate patient consent policies and data access rights.	Simplify data-sharing policies while maintaining security standards.

Source: Current research, Gagnon et al. (24), Schmitt (25).

In summary, while Nigeria, Canada, and Germany face common challenges in EHR adoption—such as usability issues, financial constraints, and privacy concerns—their healthcare structures and policy landscapes significantly influence their adoption trajectories. Each country requires tailored interventions to address its unique barriers and facilitate a smoother transition to digital health records.

In Nigeria, the biggest obstacles stem from poor infrastructure, lack of training, and weak government commitment. Without strategic investments in power supply, internet connectivity, and clinician training, EHR adoption will remain slow and ineffective. Addressing these foundational issues is crucial for ensuring that digital health systems can be implemented and sustained in hospitals across the country.

For Canada, policy inconsistencies and interoperability challenges limit the effectiveness of EHR systems. Although the country has a well-developed healthcare infrastructure, fragmented governance across provinces creates obstacles to seamless data exchange. Additionally, privacy concerns continue to generate uncertainty among patients and clinicians. To overcome these issues, national standardization efforts, clearer data governance policies, and better awareness initiatives are needed (24).

In Germany, strict privacy laws, bureaucratic inefficiencies, and a highly fragmented healthcare system have significantly slowed EHR adoption. The country’s self-governed healthcare model has contributed to prolonged delays, while public skepticism regarding data security further discourages the use of digital records. Simplifying the EHR enrollment process, strengthening governance coordination, and improving public trust through education campaigns are essential steps toward increasing adoption rates (25).

By addressing these challenges through targeted policies, infrastructure development, and user education, each country can enhance its EHR adoption process and realize the full benefits of digital health transformation. Implementing these improvements will lead to more efficient healthcare delivery, better patient outcomes, and stronger data-driven decision-making in the healthcare sector.

### 4.3 Strengths and limitations of the study

The study's strengths lie in its comprehensive qualitative approach, engaging a significant number of clinicians (326) from diverse healthcare settings, which provides rich insights into the challenges of EHR adoption. The structured interview method allowed for in-depth exploration of participants' experiences, ensuring a robust understanding of the barriers faced in implementing digital health systems. However, a limitation of the study is its focus solely on referral and university teaching hospitals within the tertiary healthcare context, which may limit the generalizability of the findings to other settings, such as primary and secondary healthcare facilities.

## 5 Conclusion

The findings from this study provide a comprehensive understanding of the multifaceted challenges hindering the successful adoption of Electronic Health Record (EHR) systems within referral and university teaching hospitals in Nigeria, revealing critical insights into the clinician experience and the systemic barriers at play. A significant observation was the pervasive lack of political will among hospital administrations, which manifested in insufficient support for digital transformation initiatives, thereby stifling progress towards effective EHR implementation. Clinicians reported a range of technical issues, including inadequate system design and infrastructural deficiencies, which not only complicated the usability of EHR systems but also contributed to system malfunctions and network difficulties. Furthermore, the study highlighted the crucial role of training, as many clinicians expressed concerns over their lack of digital literacy and the absence of comprehensive training programs, which exacerbated their resistance to change. This resistance was further fueled by overwhelming workloads that exceeded staff capacity, coupled with a lack of IT technical support to assist in the transition to digital systems. The insights derived from this research emphasize the urgent need for targeted interventions that address both the technical and human factors influencing EHR adoption, including the establishment of robust training programs, improved infrastructure, and a supportive administrative framework. By tackling these barriers, healthcare facilities can enhance the effectiveness of EHR systems, ultimately leading to improved healthcare delivery and management in Nigeria's evolving digital health landscape.

## Data Availability

The raw data supporting the conclusions of this article will be made available by the authors, without undue reservation.

## References

[1] Popov VV Kudryavtseva EV Katiyar NK Shishkin A Stepanov SI. Industry 4.0 and digitalisation in healthcare. Materials (Basel). (2022) 15(6):1–21. 10.3390/ma15062140 35329592 PMC8953130

[2] Edberg D Wendel J. Healthcare transformation: the electronic health record. In: Duckworth M, O'Donohue W, editors. Behavioral Medicine and Integrated Care. Cham: Springer (2018). p. 121–45. 10.1007/978-3-319-93003-9_7

[3] Ehrenstein V Kharrazi H Lehmann H Taylor CO. Obtaining data from electronic health records. In: Gliklich RE, Leavy MB, Dreyer NA, editors. Tools and Technologies for Registry Interoperability, Registries for Evaluating Patient Outcomes: A User's Guide, 3rd ed., Addendum 2. Rockville, MD: Agency for Healthcare Research and Quality (US) (2019). p. 52–79. Available at: https://www.ncbi.nlm.nih.gov/books/NBK551878/

[4] The Chartis Group. Organizational Readiness—the Critical Success Factor in an EHR Implementation or Merger Integration. The Chartis Group (2022). Available at: https://www.chartis.com/insights/organizational-readiness-critical-success-factor-ehr-implementation-or-merger-integration (Accessed May 08, 2024).

[5] Woldemariam MT Jimma W. Adoption of electronic health record systems to enhance the quality of healthcare in low-income countries: a systematic review. BMJ Health Care Inform. (2023) 30(1). 10.1136/bmjhci-2022-100704 37308185 PMC10277040

[6] Albano V. Organizational readiness and success of the EHR-S adoption. In: D'Atri A De Marco M Braccini A Cabiddu F, editors. Management of the Interconnected World. Rome: Physica-Verlag HD (2010). 10.1007/978-3-7908-2404-9_18

[7] Garrick R Sullivan J Doran M Keenan J. The role of the hospital in the healthcare system. Mod Hosp. (2019):47–60. 10.1007/978-3-030-01394-3_6

[8] Janssen A Donnelly C Elder E Pathmanathan N Shaw T. Electronic medical record implementation in tertiary care: factors influencing adoption of an electronic medical record in a cancer centre. BMC Health Serv Res. (2021) 21(1). 10.1186/s12913-020-06015-6 33407449 PMC7789279

[9] Boonstra A Versluis A Vos JFJ. Implementing electronic health records in hospitals: a systematic literature review. BMC Health Serv Res. (2014) 14(1). 10.1186/1472-6963-14-370 25190184 PMC4162964

[10] Derecho KC Cafino R Aquino-Cafino SL Isla A Esencia JA Lactuan NJ Technology adoption of electronic medical records in developing economies: a systematic review on physicians’ perspective. Digit Health. (2024) 10. 38222081 10.1177/20552076231224605 PMC10787531

[11] Al Meslamani AZ. Technical and regulatory challenges of digital health implementation in developing countries. J Med Econ. (2023) 26(1):1057–60. 10.1080/13696998.2023.2249757 37594521

[12] Haque M Ahsan M Rahman F Islam A. The challenges of eHealth implementation in developing countries: a literature review. IOSR J Dent Med Sci. (2019) 18:41–57. Available at: https://www.iosrjournals.org/iosr-jdms/papers/Vol18-issue5/Series-12/H1805124157.pdf

[13] Parajuli R Bohara D KC M Shanmuganathan S Mistry SK Yadav UN. Challenges and opportunities for implementing digital health interventions in Nepal: a rapid review. Front Digit Health. (2022):4. 10.3389/fdgth.2022.861019 PMC9480345 36120714

[14] Ayamolowo LB Irinoye OO Olaniyan AS. Utilization of electronic health records and associated factors among nurses in a faith-based teaching hospital, ilishan, Nigeria. JAMIA Open. (2023) 6(3). 10.1093/jamiaopen/ooad059 37545983 PMC10403426

[15] Verakki Business Solution for Africa. Healthcare in Nigeria: the promises of digital transformation. Lagos: Verakki Business Solution for Africa (2022). Available at: https://verraki.africa/ https://verraki.africa/wp-content/uploads/2022/12/Digital-Healthcare-Report_Verraki-Africa_-final-copy-for-publishing_Dec-2022-1.pdf (Accessed January 25, 2024).

[16] Tegegne MD Tilahun B Mamuye A Kerie H Nurhussien F Zemen E Digital literacy level and associated factors among health professionals in a referral and teaching hospital: an implication for future digital health systems implementation. Front Public Health. (2023) 11:1130894. 10.3389/fpubh.2023.1130894 37113180 PMC10126829

[17] Lowry SZ Ramaiah M Patterson ES Brick D Gurses AP Ozok A Integrating electronic health records into clinical workflow. Proc Int Symp Human Factors Ergon Health Care. (2014) 3(1):170–7. 10.1177/2327857914031028

[18] Koopman RJ Steege LMB Moore JL Clarke MA Canfield SM Kim MS Physician information needs and electronic health records (EHRs): time to reengineer the clinic note. J Am Board Fam Med. (2015) 28(3):316–23. 10.3122/jabfm.2015.03.140244 25957364

[19] Ajami S BagheriTadi T. Barriers for adopting electronic health records (EHRs) by physicians. Acta Inform Med. (2013) 21(2):129. 10.5455/aim.2013.21.129-134 24058254 PMC3766548

[20] Gleiss A Lewandowski S. Removing barriers for digital health through organizing ambidexterity in hospitals. J Public Health. (2021).

[21] Safi S Thiessen T Schmailzl KJ. Acceptance and resistance of new digital technologies in medicine: qualitative study. JMIR Res Protoc. (2018) 7(12):e11072. 10.2196/11072 30514693 PMC6299231

[22] Ting J Garnett A Donelle L. Nursing education and training on electronic health record systems: an integrative review. Nurse Educ Pract. (2021) 55:103168. 10.1016/j.nepr.2021.103168 34411879

[23] Akinseinde M. The challenges of implementing digital health in Nigeria (Master of science thesis). University of Salford, United Kingdom (2016). p. 1–87. Available at: https://www.researchgate.net/publication/349663009_THE_CHALLENGES_OF_IMPLEMENTING_DIGITAL_HEALTH_IN_NIGERIA (Accessed January 26, 2024).

[24] Gagnon MP Payne-Gagnon J Breton E Fortin JP Khoury L Dolovich L Adoption of electronic personal health records in Canada: perceptions of stakeholders. Int J Health Policy Manag. (2016) 5(7):425–33. 10.15171/ijhpm.2016.36 27694670 PMC4930348

[25] Schmitt T. Implementing electronic health records in Germany: lessons (yet to be) learned. Int J Integr Care. (2023) 23(1). 10.5334/ijic.6578

[26] Stoumpos AI Kitsios F Talias MA. Digital transformation in healthcare: technology acceptance and its applications. Int J Environ Res Public Health. (2023) 20(4):3407. 10.3390/ijerph20043407 36834105 PMC9963556

