# Supplementary material for: Exploring user experiences of clinicians engaged with the digital healthcare interventions across the referral and university teaching hospitals in Nigeria: a qualitative study
Source: Front Digit Health. 2025 May 29;7:1488880. doi: 10.3389/fdgth.2025.1488880 (PMC12158922; doi:10.3389/fdgth.2025.1488880)
Supplement: Supplementary file 1 [file Datasheet1.pdf]

Investigating Challenges and Limitations Bedevilling EHR-Systems Adoption across Referral and University Teaching Hospitals in Developing Economies: A Qualitative Study of the Healthcare Professionals' Perspectives

Short questions designed to help identify the challenges and limitations bedevilling ehr-systems adoption across referral and university teaching hospitals in developing economies This would help us identify areas to improve the performance of existing systems and also adequately prepare for future implementation.

Participant chosen initials (Please do not use initials that could identify you): _____________Date: ____________________

Your responses would be strictly anonymous and confidential but would also help us know the improvements the electronic health record clerking system requires.

<u>SECTION A</u>

1. **Age** (Years) __________ 2. **Gender**: Male ☐ Female ☐

3. **How long have been working at this hospital**? (Years): __________

4. **Highest education level**: High school ☐ Diploma ☐ Bachelors ☐ Masters ☐ PhD/Fellowship ☐

5. **Job title**: Doctor ☐ Nurse ☐ Health Record Officer ☐

6. **Does your daily work involve direct patient care?** Yes ☐ No ☐

7. **How often do you use a computer**? Daily ☐ A few times a week ☐ A few times a month ☐ A few times a year ☐ Never ☐

8. **How would you rate your computer skills on a scale of 1 to 5? 1**= Basic computer skills (need help with internet and email or office applications), **5**= Proficient (able to do advanced tasks such as database management or programming)

Computer skills: *circle your level*

Basic 1 — 2 — 3 — 4 — 5 Proficient

9. **Do you have any experience using electronic medical records (EHR) systems?** Yes ☐ No ☐

10. **Have you ever received any training on EHRs?** Yes ☐ No ☐

SECTION B: This section evaluates the perspective of the healthcare professional on the challenges and limitations bedevilling ehr-systems adoption across referral and university teaching hospitals in developing economies

The structured interview survey questions were as follows.

1. Do you think your hospital is Ready or Not Ready to implement an EHR? Please tick Ready or Not Ready as applicable to your hospital, and then give reasons for your answer in a short comment.

2. If there had been any computer-based project like the Electronic Health Record system attempted in your hospital, to what extent do you think it succeeded, and was it able to support doctors and nurses to store and retrieve patients' health records effectively?

3. If past Electronic Health Record projects in your hospital failed or performed below expectations and were later abandoned by the clinicians who later returned to the paper mode of storing patients' health information, what do you think caused such failure? What were the complaints made by the doctors and nurses who used these tools?

4. What challenges or limitations do you think could hamper future implementations of electronic health records projects in your hospital if not addressed?

Note: *complete survey used for this study shall be uploaded to the journal publisher along with the manuscript as supplementary material upon request*
