# Supplementary material for: Exploring user experiences of clinicians engaged with the digital healthcare interventions across the referral and university teaching hospitals in Nigeria: a qualitative study
Source: Front Digit Health. 2025 May 29;7:1488880. doi: 10.3389/fdgth.2025.1488880 (PMC12158922; doi:10.3389/fdgth.2025.1488880)
Supplement: Supplementary file 2 [file Table1.pdf]

Supplementary Material:

Supplementary Table 1. Table 2 Extended: Detailed Clinicians opinions on reasons for failed/poor performance of past electronic health records projects across the three referral/university hospitals studied

S/N	Key Themes	Frequency	Percentages (%)	Sample Quote
**1.**	**Lack of political will and poor management commitment and oversight**	**42 Entries/ Mentions**	**14.5%**	• "There is this lack of political will to get things done in our facility"<br>• "There is lack of political will to sustain the vision after several training"<br>• "Nurses have never used EHR in this hospital because its installation and kick-off had been in the pipeline for ages."<br>• "A lot of protocols with regard to red tape and political bottlenecks hinder any new project in our unit."<br>• "Getting anything done in this facility is a time-consuming process."<br>• "Our hospital is not ready to implement an EHR, from the looks of things (inability to provide materials for work)."<br>• "There had been lack of supervision of executed projects"<br>• "Sustainability: An earlier attempt was abandoned midway by the hospital administration."<br>• "Past projects never really worked."<br>• "There is concern about a lack of capacity for sustainability"<br>• "There had been poor administration of these digital implementations."<br>• "I could say that there is no strong management commitment to providing an enabling environment for such implementation to succeed."<br>• "Politics is a major challenge."<br>• "Poor commitment from the hospital administration and low computer literacy amongst staff"<br>• "The management had been too slow in commencing the implementation."

				• "Bureaucratic bottleneck in getting approvals that support the sustenance"<br>• "Inconsistency among decision-makers"<br>• "A nurse commented that, "in the past year, the computer based EHR was introduced but without effect. It wasn't implemented".<br>• "Getting anything changed in our hospital is a time-consuming process."<br>• "I think our hospital is not ready to implement an EHR because it's been over a year since we were taught about its use, but to date there has been no implementation."<br>• "The hospital management lacks the willpower to maintain EHR implementation."<br>• "The project was announced a few years ago, but since then there has been no trial and no implementation."<br>• "Lack of seriousness on the part of the hospital administration to implement EHR had been a major challenge."
**2.**	**Lack of Computer/Digital Literacy**	**29 Entries/ Mentions**	**10.0%**	• "Lack of sufficient computer skills among the staff population"<br>• "Inadequate number of computers and poor knowledge of computer operation."<br>• "Typing with the keyboard while trying to input clinical health information has remained a huge challenge for some doctors."<br>• "The computer and digital literacy of employees is quite low, and therefore the hospital might not be ready to implement an EHR."<br>• "Poor computer skill on the part of the clinical staff is high."<br>• "Time-consuming nature of inputting records using the keyboard can be boring."<br>• "Some health providers are computer illiterate, but with education, they can adapt to it.<br>• "We are ready to accept EHR, but the administrators should delay a bit for us to acquire the required computer knowledge

				and skills.<br>• “Inadequate knowledge about the EHR<br>• “It was not successful because the necessary gadgets were not in place, and some had broken down and not been repaired. Again, poor computer literacy is also a major challenge.<br>• “I think that there are a lot of factors that could cause failure of EHR implementation amongst staff, like poor knowledge of computers, a poor network, a poor EHR design not consistent with our operational protocols, and inadequate tools and equipment to support EHR implementation.<br>• “The complaints were about not being efficient with the use of computers and power failures.”<br>• “Most staff were computer illiterate and not properly trained.”<br>• “Most of the computers were not working<br>• “Poor computer literacy among the staff population is a major challenge.”<br>• “Inadequate number of computers and poor knowledge of computer operation.”<br>• “Poor commitment from the hospital administration and low computer literacy amongst staff”<br>• “The existing failings in the use of EHR in our hospital were mainly due to a lack of manpower.”
**3.**	**Poor and often lack of comprehensive training on the workings of EHR**	**27 Entries/ Mentions**	**9.3%**	• “Several clinical staff member are not yet trained on EHR.”<br>• “Following EHR training about two years ago, there has not been any sign of implementation.”<br>• “Staff needs to be trained in these areas of digital health, and incentives need to be given.”<br>• “Ignorance on the working of EHR”<br>• “The rate at which the training is going is very slow, and I don’t think with this rate they can achieve much”

				• "Incomprehensive training or sometimes no training at all, hampers the use of EHR in our facility."<br>• "The rate at which the training is moving is nothing to write home about. It has been really slow, and only a few units have received the training."<br>• "I don't think that there are enough personnel to teach how to use electronic health record systems."<br>• "The hospital is not ready because they need to train the staff first."<br>• "There was a computer-based EHR system in our hospital, but it was not successful. Poor training and lack of interest by the staff Lack of time to use the new system given the workload"<br>• "Poor training and a difficult user interface had remained a huge challenge to the existing system."<br>• "No training for nurses No proper training and the ignorance or negligence of the workers."<br>• "Inadequate computers were provided at the time, and most staff are computer illiterate and not properly trained."<br>• "Lack of maintenance, inadequate training, and network"<br>• "The challenges of inadequate training and resistance to change are major hindrances<br>• "No training yet on the EHR"<br>• "Lack of required training to sustain the project"
**4.**	**Poor Maintenance Culture**	**16 Entries/ Mentions**	**5.5%**	• "There had been persistent complaints about faulty computers."<br>• "Lack of maintenance, inadequate training, and network"<br>• "Lack of and poor maintenance of computer systems and equipment has been major problems."<br>• "Poor maintenance and management of the technology infrastructure"

				• "I would say that we are not quite ready for these digital interventions, because the system will work today and tomorrow it's off."<br>• "Lack of maintenance of the implemented system is a major challenge"<br>• "Poor maintenance of the EHR systems"<br>• "Lack of maintenance"<br>• "Poor maintenance culture has a bane to successful digitalization in our hospital"<br>• "Lack of maintenance, inadequate training, and network"
**5.**	**Poor System Design, Poor Implementation and Use-based Struggles and Challenges**	**56 Entries/ Mentions**	**19.4%**	• "The EHRs cause delays in work and patient care. Less attention to the patient and more attention to the computer screen."<br>• "The existing system had a poorly designed data interface, and access to stored data often remained difficult."<br>• "The existing EHR system slowed down clinical services."<br>• "The attempted trial of a computer-based EHR system faced many bottlenecks and is still not able to be very beneficial."<br>• "The current implementation was not well implemented."<br>• "Inadequate computers were provided at the time of implementation"<br>• "There were complaints that it takes them much time to input data into the system after attending to patients. This is understandable given that this is a new innovation."<br>• "Projects are not usually completed."<br>• "The procurement of poor and limited gadgets could not support existing systems."<br>• "Doctors are complaining that it was taking too long to enter patient data in the system."<br>• "The existing system was partially successful."<br>• "The existing system is time-consuming, and its use frustrates doctors' daily routines.<br>• "I think it was because there weren't enough computer machines supplied."<br>• "Inadequate computer systems in the

				hospital”<br>• “There had been insufficient computer systems to support existing EHR implementation”<br>• “Non-availability of hardware devices to support the EHR implementation”<br>• “Past implementation was poor.”<br>• “The existing EHR system lacks spread across the hospital clinics, wards, etc.”<br>• “The current system does not effectively support uploading images and documents.”<br>• “The EHR implementation neither failed nor was abandoned, but its implementation has been very slow.”<br>• “The information in the existing system could often not be accessible when needed.”<br>• “The system is cumbersome and time-consuming, leading to poor user satisfaction. This may hinder the readiness of staff to adopt EHR use.”<br>• “The only successful computer-based EHR in my hospital is that it keeps only the biodata of patients but does not keep the clinical information of the patients’ folders. Patients clinical data is lost equally.”<br>• “EHR here is failing not because our staff is not ready, but because the EHR system introduced to us was inefficient and not readily available, and most times it forced clinicians to queue up for someone to key in the information they had acquired.”<br>• “The current electronic system makes things very slow; typing the history is very cumbersome and slows things down.”<br>• “Limitations imposed by the software itself (not being able to book theatre using the EHR) are problems too”<br>• “Also inefficient use by some of the workers, as some important information was still not accessible when logged in.”<br>• “The existing system poses a lot of difficulty in accessing patient medical records online.<br>• “Difficulty in retrieving stored records.”

				• "The complaints by the doctors were about a lack of time to key in the documents."<br>• "The current EHR was poorly designed, not consistent with our operational protocols was our major issue."
**6.**	**Infrastructure: System breakdown and network issues**	**39 Entries/ Mentions**	**13.5%**	• "Poor network connectivity and a low number of staff had been the bane of HER implementation in our facility."<br>• "Poor and frustrating internet and network speeds were major challenges."<br>• "Poor network has remained a huge challenge here"<br>• "There had been persistent network problem."<br>• "There is this challenge of constant network failure while using the system"<br>• "Network disruption is also a problem."<br>• "There is always poor or incomplete documentation due to system failures"<br>• "System breakdown and interrupted network services."<br>• "The computers were usually faulty and not fixed."<br>• "Most of the computers were not working effectively,"<br>• "No network to start and finish what you are typing is a major challenge."<br>• "Network failure was a major challenge."<br>• "The failure of the HER was a result of network problems, which fluctuate."<br>• "A major challenge was the poor network"<br>• "I think the major cause of negligence or ignoring the HER system could be poor network connections, which can delay the speed at which the patient's data should be handled."<br>• "Sometimes network issues have been a huge challenge."<br>• "I think that there are a lot of factors that could cause failure of HER implementation amongst staff, like poor knowledge of computers, a poor network and inadequate

				tools and equipment to support HER implementation."<br>• "Recurrent and persistent network failures have remained a challenge."<br>• "Challenges with poor networks have been a bane in the full utilisation of past EHR systems."<br>• "Network failure was a major challenge<br>• "Poor network is a challenge."<br>• "Poor internet/network has been a major problem"<br>• "Network speed is so slow that retrieving uploaded patient health information can sometimes be difficult."
**7.**	**Poor staff commitment, reluctance and Resistance to Change**	**21 Entries/ Mentions**	**7.3%**	• "Again, some people still don't want to embrace change."<br>• "The hospital appears not ready because the paper method or mode of storing patient's health information has been the only existing method in my hospital, notwithstanding the attempt made at introducing EHR in our hospital."<br>• "Poor commitment and cooperation from staff"<br>• "A seeming challenge may be that it appears that the doctors and nurses have adapted to the paper mode of storing patients health information and this could affect their attitude towards the digital mode."<br>• "Most people were not interested. Many were averse to change."<br>• "Also, the availability of paper (still in circulation) encouraged the return to paper mode. Also inefficient use by some of the workers, as some important information was still not accessible when logged in."<br>• "Resistance to change from the staff population"<br>• "Resistance to change amongst staff or poor staff dedication to the EHR project"<br>• "Also, the availability of paper (still in circulation) encouraged the return to paper

				mode"<br>• "Past efforts did not last because of a lack of cooperation by the staff (nurses) and the lack of doctors to implement them."
**8.**	**Poor staff motivation**	**4 Entries/ Mentions**	**1.4%**	• "Poor support system and a lack of staff motivation."<br>• "There is lack of motivation amongst staff"<br>• "The current staff population is poorly motivated."
**9.**	**Poor sensitization**	**2 Entries/ Mentions**	**0.7%**	• "I think seminars and workshops should be organised to enlighten nurses and doctors on the idea of EHRs and how to use them properly."<br>• "There has not been any widespread sensitization about EHR from hospital management."
**10.**	**Poor power supply**	**16 Entries/ Mentions**	**5.5%**	• "Inadequate supply of power."<br>• "Management should improve the network and power supply."<br>• "The complaints were that there was no chance, time, or even power supply to support the training and implementation."<br>• "Power failure and a low-quality computer that will work today and will not work tomorrow may cause everything you type to disappear once the light is out. Or everything will hang when you want to submit what you have typed."<br>• "Not that HER has failed in my hospital, but they face some challenges like network failure and power failure."<br>• "Power failure (an epileptic power supply) was really a major challenge."<br>• "Challenges with epileptic power supplies were indicated as a major challenge."<br>• "Epileptic power supply"<br>• "The complaints were about not being efficient with the use of computers and power failures."
**11.**	**Excessive Workload**			• "High doctor-to-patient ratio, which left no

	**vis-à-vis Poor Staff Strength**	**31 Entries/ Mentions**	**10.7%**	time to manage the EHR."<br>• "The workload in the hospital is already high, and introducing an EHR would further compound everything."<br>• "We usually have plenty of patients in the clinic, so typing on the keyboard under such pressing circumstances slows things down."<br>• "Insufficient human and material resources to see it to its logical conclusion"<br>• "Large number of patients to be seen, few nurses doing the job, and high nurse-to-patient ratio."<br>• "More doctors would be needed to run the EHR as the new culture would be time-consuming and less would be achieved by doctors using the EHR."<br>• "Poor staff strength is a huge challenge in our facility."<br>• "Past efforts were not successful because staff strength is limited."<br>• "This hospital is not ready because of the high nurse-to-patient ratio and increased workload. The waiting time for the patients will be long, and the agitation of the patients will disrupt a smooth working environment."<br>• "The pressure of work made doctors and nurses inefficient in their use of EHR."<br>• "Inadequate staffing, a huge number of patients seen, nurses struggling to finish their procedures before handing over to the next shift."<br>• "The hospital is not ready to implement EHR because manpower is low and patient turnout is high. So the use of EHRs could be time-consuming in clinics."<br>• "Due to the high workload, typing on the keyboard under such circumstances for each patient can be time-consuming."<br>• "The existing EHR in our hospital is not effective because of the patient load in most government facilities; it is usually more time-consuming. The physician takes more time to see fewer patients. "

				• "We are not ready due to our enormous workload."<br>• "Patient load and doctor load are hindrances to efficient use of EHR."<br>• "It is time-consuming inputting records relative to the workload."<br>• "The patient/doctor ratio is high, which implies excess workload."
**12.**	**Funding Challenge**	**3 Entries/ Mentions**	**1.0%**	• "Financial and funding constraints to implement the project"<br>• "Insufficient take-off capital"<br>• "The hospital is ready, but their readiness may be hindered by finances, for it is difficult for the government to embark on projects. "
**13.**	**Lack of IT Technical Support Staff to support implementation**	**3 Entries/ Mentions**	**1.0%**	• "IT support staff is not always available. "<br>• "Lack of IT personnel to assist in the event of any challenges"<br>• "Lack of personnel to handle such implementation"
		**Total = 289 Entries**		
