# Supplementary material for: Exploring user experiences of clinicians engaged with the digital healthcare interventions across the referral and university teaching hospitals in Nigeria: a qualitative study
Source: Front Digit Health. 2025 May 29;7:1488880. doi: 10.3389/fdgth.2025.1488880 (PMC12158922; doi:10.3389/fdgth.2025.1488880)
Supplement: Supplementary file 3 [file Supplementaryfile1.pdf]

COREQ (COnsolidated criteria for REporting Qualitative research) Checklist

A checklist of items that should be included in reports of qualitative research. You must report the page number in your manuscript where you consider each of the items listed in this checklist. If you have not included this information, either revise your manuscript accordingly before submitting or note N/A.

Topic	Item No.	Guide Questions/Description	Reported on Page No.
**Domain 1: Research team and reflexivity**			
Personal characteristics			
Interviewer/facilitator	1	Which author/s conducted the interview or focus group?<br>**OUS was responsible for the distribution of the structured interview questionnaire.**	Page 7
Credentials	2	What were the researcher's credentials? E.g. PhD, MD<br>**OO, NP and FB are PhDs and full Professors in Computing and Medicine/Healthcare; OWO and OO are also MDs and TS is a PhD**	These details were not reported
Occupation	3	What was their occupation at the time of the study?<br>**OWO and OO are Consultant Orthopaedic Surgeons and Lecturers/Professors at the Colleges of Medicine (Medical School) of the Tertiary/University Teaching Hospitals studied.**<br>**NP is a Professor of Computing in Berlin, Germany. FB is a Professor of Medical Informatics at a University of Medicine in Berlin, and TS is a Lecturer in Digital Health at the University of Medicine in Berlin. OUS is a Doctoral Candidate in Berlin and under the supervision of NP, TS and FB**	These details were not reported
Gender	4	Was the researcher male or female?<br>**All researchers are Male**	Not reported
Experience and training	5	What experience or training did the researcher have?<br>**Researchers coming from computing background (OUS, TS, NP and FB) are practically experienced in human-centered computing involving human participants, while the researchers coming from medicine and healthcare (FB, OO and OWO) are also experienced in clinical research, which also involves human participants. So all have formal training and experience in the conduct of qualitative research**	These details were not reported
Relationship with participants			
Relationship established	6	Was a relationship established prior to study commencement?<br>**Yes, there was established relationship with some clinicians who provided contacts and spoke to many to enable us gain the trust of stakeholders before the research began. Most of the participants in the research were also colleagues to some members of the research team, working in the same teaching hospitals.**	Not reported
Participant knowledge of the interviewer	7	What did the participants know about the researcher? e.g. personal goals, reasons for doing the research<br>**All participants were first educated and convinced on the goals of the research before their involvement.**	Not reported
Interviewer characteristics	8	What characteristics were reported about the inter viewer/facilitator? e.g. Bias, assumptions, reasons and interests in the research topic<br>**The goals, reasons and interest of the interviewer in the research topic were reported**	Page 1, 6 and 8
**Domain 2: Study design**			

Theoretical framework			
Methodological orientation and Theory	9	What methodological orientation was stated to underpin the study? e.g. grounded theory, discourse analysis, ethnography, phenomenology, content analysis<br>**This study involved one research paradigms, like qualitative research paradigms.**	Page 6 and 10
Participant selection			
Sampling	10	How were participants selected? e.g. purposive, convenience, consecutive, snowball<br>**Purposive sampling was used**	Page 7
Method of approach	11	How were participants approached? e.g. face-to-face, telephone, mail, email<br>**All participants in this study were physically approached, Face-to-face**	Page 7
Sample size	12	How many participants were in the study?<br>**326 participants were particularly involved**	Page 11

Non-participation	13	How many people refused to participate or dropped out? Reasons?<br>**Not Applicable**	Not Applicable
Setting			
Setting of data collection	14	Where was the data collected? e.g. home, clinic, workplace<br>**All data were collected at Clinic**	Page 7
Presence of nonparticipants	15	Was anyone else present besides the participants and researchers?<br>**Yes, the clinicians often did have colleagues present**	Not Reported
Description of sample	16	What are the important characteristics of the sample? e.g. demographic data, date<br>**Please see Table 1**	Page 11, Table 1
Data collection			
Interview guide	17	Were questions, prompts, guides provided by the authors? Was it pilot tested?<br>**The interviews were structured; using structured interview survey to elicit the healthcare professionals' perspective.**	Page 7, 8, 9, 11
Repeat interviews	18	Were repeat interviews carried out? If yes, how many?<br>**The interview was administered as a paper questionnaire and the clinicians responded at a time convenient for them. And later the questionnaires were retrieved**	Page 7
Audio/visual recording	19	Did the research use audio or visual recording to collect the data?<br>**No**	Not Applicable
Field notes	20	Were field notes made during and/or after the interview or focus group?<br>**We used paper-based structured interview survey.**	Not Reported
Duration	21	What was the duration of the interviews or focus group?<br>**The time for distribution and collection was about 4 weeks at each of the referral and teaching hospital.**	Not Reported
Data saturation	22	Was data saturation discussed?<br>**We had a target of 400 participants and we had 326 respond; but that was when had collected all information necessary for our study.**	Not Reported
Transcripts returned	23	Were transcripts returned to participants for comment and/or correction?	Not Applicable
**Topic**	**Item No.**	**Guide Questions/Description**	**Reported on Page No.**
		**No audio and video were used during study**	

**Domain 3: analysis and findings**			
Data analysis			
Number of data coders	24	How many data coders coded the data?<br>**1 coder**	Page 9
Description of the coding tree	25	Did authors provide a description of the coding tree?	Not Reported
Derivation of themes	26	Were themes identified in advance or derived from the data?<br>**Themes were identified from the data collected**	Not Reported
Software	27	What software, if applicable, was used to manage the data?<br>**It was manually managed**	Page 7
Participant checking	28	Did participants provide feedback on the findings?<br>**No**	Not Applicable
Reporting			
Quotations presented	29	Were participant quotations presented to illustrate the themes/findings? Was each quotation identified? e.g. participant number<br>**The participants quotations were presented under each identified theme.**	Not Reported
Data and findings consistent	30	Was there consistency between the data presented and the findings?<br>**Yes**	Yes
Clarity of major themes	31	Were major themes clearly presented in the findings?<br>**Yes**	Yes
Clarity of minor themes	32	Is there a description of diverse cases or discussion of minor themes?<br>**Themes were never preset before the structured interview. Themes only emerged from the interview survey.**	Not Reported

Developed from: Tong A, Sainsbury P, Craig J. Consolidated criteria for reporting qualitative research (COREQ): a 32-item checklist for interviews and focus groups. *International Journal for Quality in Health Care*. 2007. Volume 19, Number 6: pp. 349 – 357
